# Increased Amplitude of Low Frequency Fluctuations but Normal Hippocampal-Default Mode Network Connectivity in Schizophrenia

**DOI:** 10.3389/fpsyt.2015.00092

**Published:** 2015-06-24

**Authors:** Maureen McHugo, Baxter P. Rogers, Pratik Talati, Neil D. Woodward, Stephan Heckers

**Affiliations:** ^1^Department of Psychiatry, Vanderbilt University, Nashville, TN, USA; ^2^Institute of Imaging Sciences, Vanderbilt University, Nashville, TN, USA; ^3^Vanderbilt Brain Institute, Vanderbilt University, Nashville, TN, USA

**Keywords:** schizophrenia, hippocampus, connectivity, biomarkers, neuroimaging, fMRI

## Abstract

**Background:**

Clinical and preclinical studies have established that the hippocampus is hyperactive in schizophrenia, making it a possible biomarker for drug development. Increased hippocampal connectivity, which can be studied conveniently with resting state imaging, has been proposed as a readily accessible corollary of hippocampal hyperactivity. Here, we tested the hypothesis that hippocampal activity and connectivity are increased in patients with schizophrenia.

**Methods:**

Sixty-three schizophrenia patients and 71 healthy control subjects completed a resting state functional magnetic resonance imaging scan. We assessed hippocampal activity with the amplitude of low frequency fluctuations. We analyzed hippocampal functional connectivity with the default mode network using three common methods: group and single subject level independent component analysis, and seed-based functional connectivity.

**Results:**

In patients with schizophrenia, we observed increased amplitude of low frequency fluctuations but normal hippocampal connectivity using independent component and seed-based analyses.

**Conclusion:**

Our results indicate that although intrinsic hippocampal activity may be increased in schizophrenia, this finding does not extend to functional connectivity. Neuroimaging methods that directly assess hippocampal activity may be more promising for the identification of a biomarker for schizophrenia.

## Introduction

Neuroimaging and postmortem studies indicate that function and structure of the hippocampus are abnormal in schizophrenia (1). Current models posit that NMDA-receptor hypofunction (2, 3) or a deficit of GABAergic interneurons (4, 5) leads to a hyperactive hippocampus in schizophrenia (1, 5, 6). The most compelling evidence for hippocampal hyperactivity in schizophrenia comes from studies of absolute brain activity, measured *in vivo* using cerebral blood flow (CBF) and cerebral blood volume (CBV) methods (7–13). The degree of hippocampal hyperactivity has been linked to overall psychopathology (14) and positive symptoms (15, 16), and resolves with antipsychotic treatment (17). This has led to the proposal that hippocampal hyperactivity is a biomarker that can guide the development of new treatments for schizophrenia (6).

Resting state fMRI (RS-fMRI) has been used to support the proposal that hippocampal hyperactivity may be a biomarker for schizophrenia. RS-fMRI measures spontaneous temporal changes in the blood oxygen level dependent (BOLD) response. RS-fMRI has been most commonly used to examine intrinsic functional connectivity, the strength of co-activation between different brain regions over time, rather than activity within a region *per se*. Tregellas and colleagues (12) recently identified increased functional connectivity between the hippocampus and other brain regions, particularly those in the default mode network. The hippocampus is an accessory component of the default mode network and is most often associated with episodic memory and prospective mental scene construction (18). Both hippocampal hyperactivity and default mode network dysconnectivity have been linked to memory deficits and positive symptoms in schizophrenia (1, 19).

While intriguing, there are several issues that warrant additional exploration. First, in the analysis used by Tregellas et al., estimation of individual subject connectivity relies on back reconstruction from group level results (20). Hippocampal connectivity or activity can become a surrogate endpoint in clinical trials only if it can be measured at the individual subject level. Second, although increased functional connectivity was reported by one other study (21), more studies have reported decreased (22, 23) or normal (24–29) functional connectivity of the hippocampus in schizophrenia. The conflicting results may be due to variable methodology used across studies [data driven independent component analysis (ICA) versus hypothesis-driven seed-based analyses]. Finally, functional connectivity differences can be due to abnormal activation of multiple brain regions. To test for functional connectivity differences due to hippocampal hyperactivity, it is necessary to study intrinsic activity within the hippocampus itself, such as the amplitude of low frequency fluctuations (ALFF) (30). ALFF is correlated with task-related activation in task positive regions and with task-related deactivation in regions of the default mode network (31, 32). Thus, it serves as a useful marker of intrinsic brain activity. Two recent studies have found increased hippocampal ALFF in schizophrenia (33, 34).

Taken together, the evidence for hippocampal hyperactivity in schizophrenia is compelling (6), but the finding of increased intrinsic hippocampal functional connectivity in chronic schizophrenia (12) requires confirmation. The current study had two goals: (1) to determine whether increased hippocampal activity and connectivity could be replicated in a large sample with the same methodology used in previous studies (12, 33) and (2) to examine whether hippocampal hyperconnectivity is present using measures of intrinsic connectivity better suited to analysis at the individual subject level. We measured BOLD signal fluctuations within the hippocampus using ALFF and fractional ALFF. Fractional ALFF was included because it may be less sensitive to physiological noise than ALFF (35). Two types of ICA were performed in which mean independent component value was extracted from hippocampal regions of interest. *Group level* ICA was used to determine if the previous finding of hippocampal hyperconnectivity in schizophrenia (12) was replicated in our data. Although other methods may be superior for group ICA (36), such methods still derive connectivity information for individual subjects from group level data. We therefore also measured *individual subject* ICA, which allows direct assessment of connectivity at the individual level and avoids the estimation of individual subject connectivity using back reconstruction that is required for group level ICA (20). Additionally, we carried out seed-based connectivity analysis between the hippocampus and several key nodes of the default mode network.

**Table 1:** **Participant demographic and clinical characteristics**.

	Schizophrenia*n* = 63	Control*n* = 71	*p-*Value
Age (years)	37.29 (11.57)	35.21 (10.84)	0.29
Gender (female/male)	27/36	33/38	0.67
Race (white/black/other)	35/23/5	46/21/4	0.54
Parental education (years^a^)	13.51 (2.73)	13.87 (2.45)	0.47
Handedness (right/left)	53/10	64/7	0.30
Premorbid IQ – WTAR	97.46 (14.34)	108.79 (12.69)	<0.001
PANSS			
Positive subscale	20.30 (6.88)		
Negative subscale	14.95 (6.88)		
General subscale	32.81 (8.06)		
Chlorpromazine equivalents^b^	566.41 (265.39)		
Smokers/non-smokers	37/26	14/57	<0.001

*All values are mean (SD) unless otherwise specified*.

*^a^Parental education levels were available for 56 patients and 53 controls*.

*^b^Chlorpromazine equivalents were available for 59 patients*.

## Materials and Methods

### Participants

Sixty-three patients with chronic schizophrenia and 71 healthy controls participated in the study (Table 1). Patients were recruited from the psychiatric inpatient and outpatient clinics of the Vanderbilt University Psychotic Disorders Program as part of an ongoing study of the neurobiology of psychosis. Age- and gender-matched healthy controls were recruited from the surrounding community using email advertisements. All participants were assessed with the Structured Clinical Interview for DSM-IV (37) and the Wechsler Test of Adult Reading (38) to estimate premorbid IQ. Participants were excluded for significant medical and neurological illness, head injury, estimated premorbid IQ <70 or alcohol, or substance abuse within the past 1 month. Control participants were further excluded for psychotropic drug use, psychiatric illness, or a first-degree relative with a history of psychotic illness. Parental education was available on a subset of 56 patients and 53 controls. Patients and controls did not differ with respect to age, race, gender, or parental education, but control participants had higher premorbid IQ and there were more smokers in the patient group (Table 1). However, tobacco use did not interact with group in any of the primary measures of hippocampal connectivity or ALFF (Group X Smoking Status ANOVAs, all *p*’s > 0.05). Antipsychotic dosage (CPZ equivalents) was not associated with primary measures of hippocampal connectivity or ALFF (Pearson correlations, all *p*’s > 0.05). The Vanderbilt University Institutional Review Board approved the study. All participants provided written informed consent and were compensated financially for their time.

### fMRI data acquisition

Structural and functional scans were acquired on a 3T Philips Achieva scanner (Philips Healthcare, Inc., Best, The Netherlands). High-resolution structural images were acquired with a 3D T1-weighted sequence [echo time (TE) = 3.7 ms; repetition time (TR) = 8.0 ms; field of view (FOV) = 256 mm^2^; number of slices = 170; slice thickness = 1.0 mm; gap thickness = 0.0 mm]. For the resting state scan, participants were instructed to remain quiet with their eyes closed but to not fall asleep. An experimenter verbally confirmed that participants stayed awake immediately following the resting state scan. Two hundred and three functional images were collected using a T2*-weighted EPI sequence (TE = 35 ms; TR = 2000 ms; flip angle = 79°; FOV = 240 mm^2^; in-plane resolution = 3.0 mm^2^; slice thickness = 4.0 mm; gap thickness = 0.4 mm; number of slices = 28).

### Structural and functional MRI data analysis

Structural and functional data were preprocessed using SPM8^1^ and Matlab (The MathWorks, Inc., Natick, MA, USA). Structural data were segmented into gray matter, white matter, and cerebrospinal fluid using the Voxel-Based Morphometry Toolbox^2^ (Version 8.0). Functional images were slice-time corrected, realigned to the mean image, co-registered with the native space structural data, and normalized to MNI space. These preprocessed images were used as input to the independent component and seed-based connectivity analyses.

#### Region of interest definition

Regions of interest (ROIs) were defined for use in the independent component and seed-based connectivity analyses described below. ROIs for the default mode network were taken from the Wake Forest University PickAtlas [version 2.4; (39)] and included the bilateral hippocampus, precuneus/posterior cingulate cortex, lateral parietal cortex (angular gyrus), and medial prefrontal cortex (including superior frontal gyrus and medial orbital gyrus). The default mode ROIs were merged into a single network template mask for use in the independent component analyses. For the seed-based connectivity and ALFF analyses, participant-specific manually traced hippocampal ROIs were used instead of the PickAtlas ROI. Bilateral hippocampal ROIs were manually traced on each participant’s native space T1-weighted structural image (40) and normalized to MNI space using each subject’s warping parameters derived from the anatomical normalization step described above. Default mode seed ROIs were the same as those used for ICA.

#### ALFF Analysis

Voxelwise amplitude of low frequency fluctuation analysis was carried out with AFNI’s 3dRSFC (41). The ALFF for each voxel was calculated by first removing linear and quadratic trends from the time-series, band-pass filtering the time-series (0.01–0.1 Hz), converting to the power spectrum using a fast Fourier transform, and taking the average of the square root of the power in the range 0.01–0.1 Hz. This value was divided by the global within-brain mean ALFF. Because ALFF may be influenced by physiological noise, we also examined fractional ALFF [fALFF, (35)]. fALFF was calculated in 3dRSFC as the ratio of the power in the low frequency range relative to the whole frequency range. fALFF for each voxel was divided by global within-brain mean fALFF. The mean ALFF and fALFF values were extracted from each hippocampal ROI and analyzed using two-tailed, Welch’s unpaired *t*-tests.

### Independent component analysis – group level

Group specific ICA was carried out to determine whether we could replicate previous findings with identical methods (12). This analysis used GIFT software^3^ (Group ICA of fMRI Toolbox). Time series were scaled to a mean of 100 on a voxel-wise basis. Each subject’s full spatiotemporal data set (time by voxel matrix) was reduced to 35 principal components. These were concatenated within each group (control or patient) and a second reduction to 20, 30, or 40 components was performed for each group matrix. Component ordering was tracked so that subject-specific component images could be back-reconstructed from the group independent components (20). Candidates for the default mode network component were identified for each group by computing the correlation between each independent component spatial map and the network template mask. The voxel values of the default mode network component were averaged across the entire hippocampus in each subject-specific component image. These average hippocampal default mode network loadings were then compared between groups for the three analyses (having 20, 30, or 40 independent components).

#### Independent Component Analysis – Individual Subjects

Single-session ICA was performed using FEAT (fMRI Expert Analysis Tool, version 6.0) and MELODIC [Multivariate Exploratory Linear Decomposition into Independent Components, version 3.12; (42)] as part of FSL^4^ (FMRIB’s Software Library). The following steps were applied to the preprocessed fMRI time-series before ICA: grand-mean intensity normalization, high-pass temporal filtering (sigma = 50.0 s), voxel-wise de-meaning, and variance normalization. Single-session ICA was carried out on each participant’s fMRI time-series multiple times: once with the number of components automatically estimated using Bayesian model selection and then with the number of components specified at 20, 30, 40, 50, 60, or 70 (42, 43). To identify the default mode network component for each participant, we selected the unthresholded component map with the highest correlation to the default mode network mask. The mean independent component value was extracted from the hippocampal ROIs (referred to as hippocampal connectivity) and analyzed using R (44). Our primary analysis was of hippocampal connectivity determined with an automatically estimated number of components and was analyzed with two-tailed, Welch’s unpaired *t*-tests. The follow-up analysis of the effect of number of components was analyzed using repeated measures ANOVAs with Group as a between subject factor and Hemisphere and Number of Independent Components as a within subject factor. Degrees of freedom were Greenhouse-Geisser corrected for violations of sphericity. Significant interactions were further examined with pairwise Welch’s *t*-tests Bonferroni-corrected for multiple comparisons.

#### Seed-Based Connectivity Analysis

ROI–ROI functional connectivity analyses were conducted with the CONN-fMRI toolbox [version 13p; (45)]. Participant-specific temporal confounds, including the realignment parameters derived from motion correction, and their first temporal derivatives, and the white matter and cerebrospinal fluid signals, were removed from the preprocessed fMRI time series. Importantly, nuisance regressors for white matter and CSF were derived from each subject’s white matter and CSF segmentations using the anatomical component-based noise reduction method (aCompCor), as implemented in the CONN-fMRI toolbox. The aCompCor method has been shown to be effective at reducing the effects of head movement on functional connectivity estimates (46). Finally, the resulting time-series was band-pass filtered (0.01–0.1 Hz). ROI–ROI connectivity for each participant was calculated as the bivariate correlation between the mean time courses of each hippocampal ROI (left and right) and each default mode ROI (medial prefrontal cortex, posterior cingulate/precuneus, lateral parietal cortex) and analyzed using R (44). Correlation values were converted to *z*-statistics using Fisher’s *z* transform. Connectivity with the default mode network was calculated separately for each hippocampal ROI as the mean *z*-statistic between the hippocampus and the default mode network ROIs. Group analyses of mean connectivity were carried out for each hippocampal ROI using two-tailed, Welch’s unpaired *t*-tests with an alpha of 0.05.

## Results

We tested the hypothesis of hippocampal hyperactivity in schizophrenia with four complementary methods: ALFF, group ICA, single subject ICA, and seed-based connectivity.

### ALFF analysis

**Figure 1:**
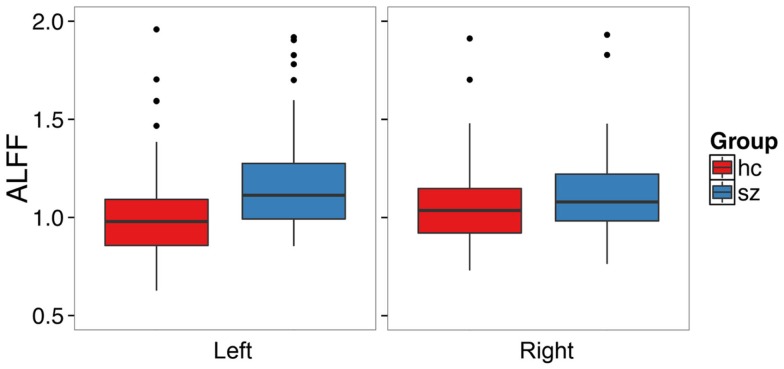
**Patients with schizophrenia have increased amplitude of low frequency fluctuations in the left hippocampus**. The boxplots show the median, interquartile range, and outliers. The whiskers extend to the lowest value within 1.5 times the quartiles.

Schizophrenia patients had significantly increased ALFF in the left hippocampus (*t*_1,126.68_ = 3.72, *p* = 0.0003, Figure 1, Table 2) and at trend level in the right hippocampus (*t*_1,129.25_ = 1.88, *p* = 0.06). We confirmed this finding with fALFF (left: *t*_1,131.85_ = 1.98, *p* = 0.05; right: *t*_1,129.60_ = 1.27, *p* = 0.21). We further analyzed *Z*-standardized ALFF and fALFF in order to control the effects of inter-individual differences in head motion (47). The finding of greater left hippocampal ALFF and fALFF in schizophrenia patients remained significant (ALFF: *t*_1,126.10_ = 3.60, *p* = 0.0005; fALFF: *t*_1,131.66_ = 2.00, *p* = 0.05).

**Table 2:** **Means and SDs of ALFF and fALFF for the hippocampus in each group**.

Hemisphere	Method	Schizophrenia	Control
Left	ALFF	1.19 (0.27)	1.02 (0.24)
	fALFF	0.89 (0.07)	0.87 (0.07)
Right	ALFF	1.12 (0.22)	1.06 (0.21)
	fALFF	0.90 (0.07)	0.88 (0.07)

### Independent component analysis – group level

**Figure 2:**
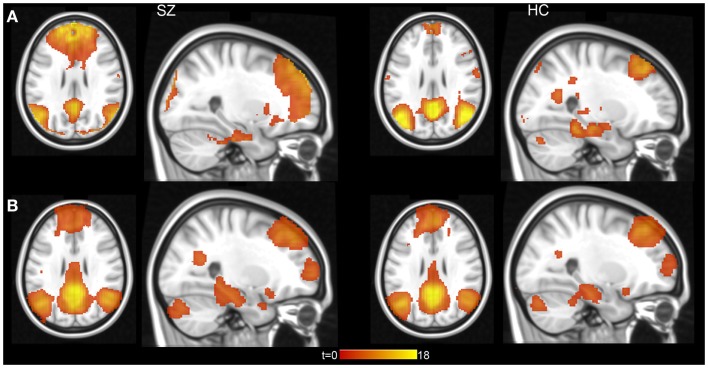
**Hippocampal connectivity with the default mode network in schizophrenia patients and healthy controls**. **(A)** Results from group level ICA 20 component analysis. **(B)** Results from individual subject level ICA analysis with automatically determined number of components. Statistical maps displayed at *p* < 0.001, uncorrected.

We used group ICA to study intrinsic hippocampal connectivity in patients relative to controls (Figure 2A). At least two independent components were reasonable candidates for the default mode network based on the correlation between their spatial maps and the template (Table 3). The best matching component varied between one characteristic of the typical default mode network, with weighting in the anterior medial frontal lobe and the lateral parietal lobe and a component heavily weighted to posterior cingulate and precuneus.

**Table 3:** **Correlation of independent component (IC) maps from group ICA analysis with default mode network template**.

Number of ICs in analysis	IC map	Schizophrenia	Control
20	1	0.40	0.31
	2	0.27	0.30
30	1	0.36	0.32
	2	0.34	0.32
40	1	0.35	0.34
	2	0.29	0.22

**Figure 3:**
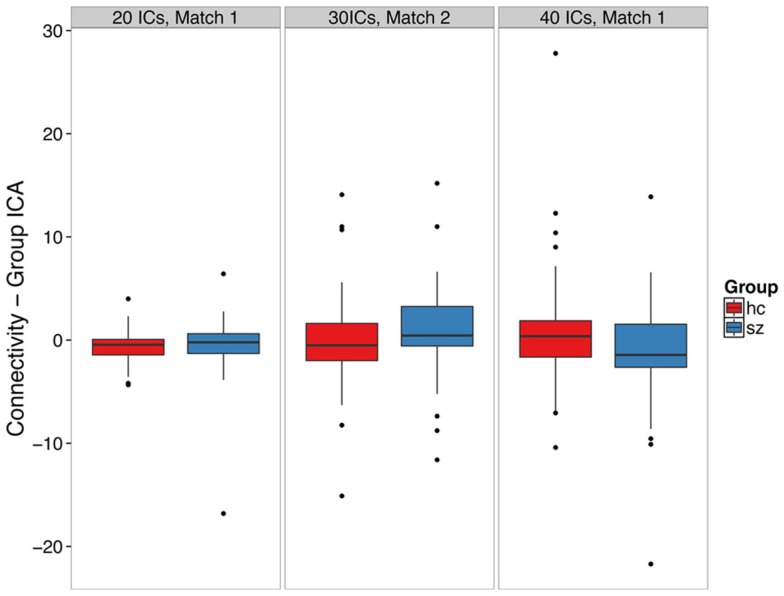
**Group ICA-derived left hippocampal hyperconnectivity depends on analysis parameters**. The boxplots show the median, interquartile range, and outliers. The whiskers extend to the lowest value within 1.5 times the quartiles.

We did not find clear evidence of hippocampal hyperconnectivity in schizophrenia patients using group ICA (Figure 3). Different processing choices yielded all possible results: no difference in connectivity between patients and controls (e.g., component 1 from the 20 component ICA), greater connectivity for patients (e.g., component 2 from the 30 component ICA), or greater connectivity in controls (e.g., component 1 from the 40 component ICA).

### Independent component analysis – individual subjects

**Figure 4:**
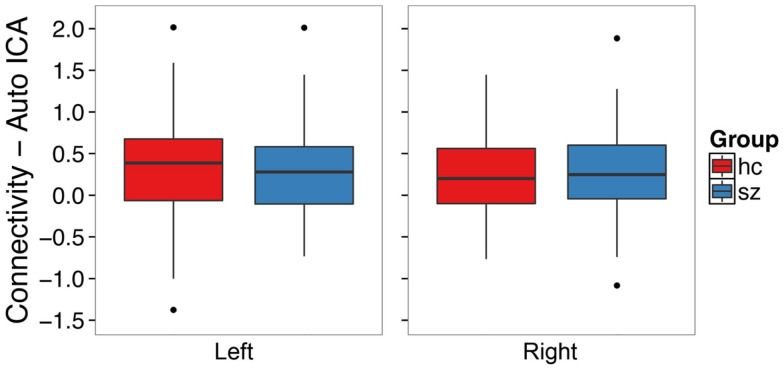
**Functional connectivity with the default mode network from single subject level ICA with automatic component estimation does not differ between schizophrenia patients and healthy controls in the left or right hippocampus**. The boxplots show the median, interquartile range, and outliers. The whiskers extend to the lowest value within 1.5 times the quartiles.

We conducted an ICA in which the number of components in the data was automatically identified in a data-driven manner [Figure 2B (42)]. Contrary to our hypothesis, schizophrenia patients did not exhibit hippocampal hyperconnectivity (Figure 4; left: *t*_132_ = -0.33, *p* = 0.74; right: *t*_129_ = 0.28, *p* = 0.78).

**Figure 5:**
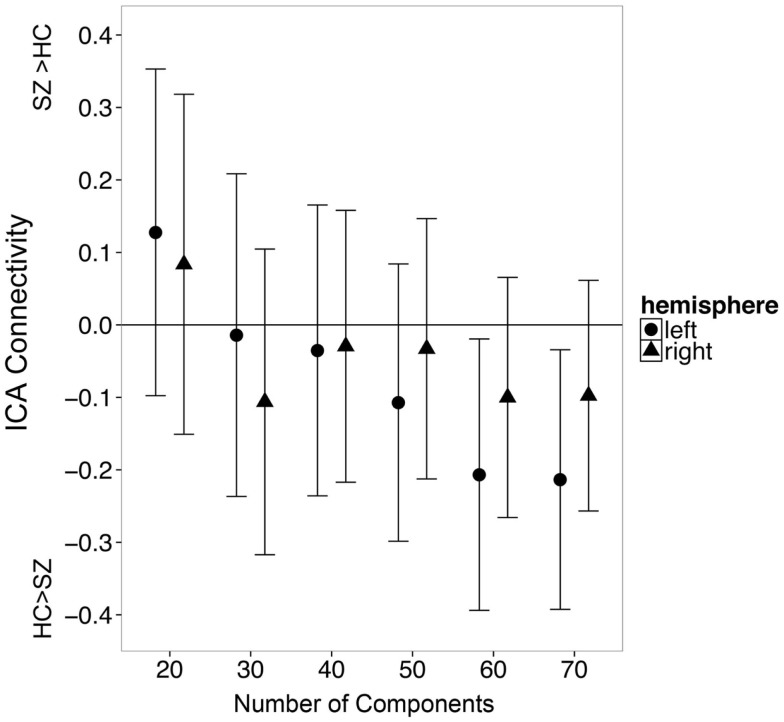
**The number of independent components specified during single subject ICA influences apparent group differences in functional connectivity between the hippocampus and the default mode network**. Error bars indicate 95% confidence interval of the between group difference in connectivity.

In secondary analyses, we examined whether varying the number of independent components would affect the observed results. We found that the number of components influenced which group exhibited greater hippocampal connectivity at a trend level [Figure 5; Group X Hemisphere X Number of Components interaction: *F*_(3.9, 514.8)_ = 2.29, *p* = 0.06]. We carried out follow-up unpaired *t*-tests separately for the left and right hippocampus at each model order (20–60 components). All tests failed to meet significance after correction for multiple comparisons (all *p*’s > 0.008). However, there was a trend for controls to have greater hippocampal connectivity than patients with 60 independent components (*t*_125_ = 2.18, *p* = 0.03).

### Seed-based connectivity analysis

**Figure 6:**
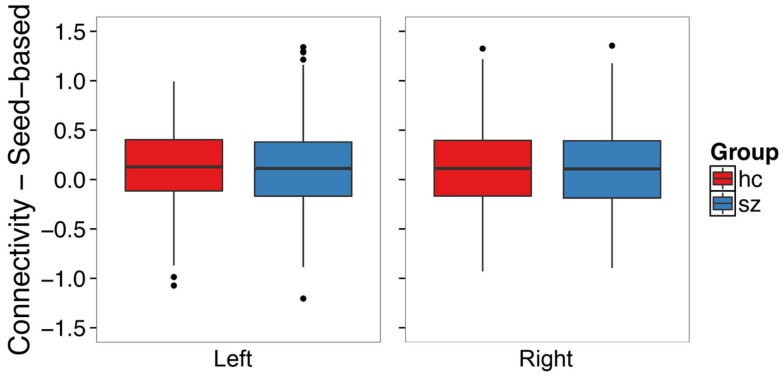
**Seed-based functional connectivity with the default mode network does not differ between schizophrenia patients and controls in the left or right hippocampus**. The boxplots show the median, interquartile range, and outliers. The whiskers extend to the lowest value within 1.5 times the quartiles.

We examined whether schizophrenia is associated with hyperconnectivity of the hippocampus and the default mode network using seed ROI–ROI functional connectivity. We found no evidence of hyperconnectivity in the schizophrenia group in the left (*t*_120.30_ = -0.49, *p* = 0.62) or right (*t*_122.19_ = 0.16, *p* = 0.88) hippocampus (Figure 6). Connectivity results were unchanged after controlling for median framewise displacement (left: *F*_1,31_ = 0.25, *p* = 0.62; right: *F*_1,31_ = 0.13, *p* = 0.71).

## Discussion

We tested the hypothesis that schizophrenia patients exhibit increased intrinsic hippocampal activity (6, 12). Patients had greater amplitude of low-frequency fluctuations (ALFF) in the left hippocampus. Our result is consistent with the hypothesis of hippocampal hyperactivity in schizophrenia. This finding adds to the growing body of evidence for greater hippocampal ALFF in chronic schizophrenia (33, 34) and fits with results from contrast-enhanced imaging (7–9, 11, 13, 16). However, we did not find evidence that this extends to hyperconnectivity using either ICA or seed-based analyses.

The finding of increased hippocampal ALFF contrasts with the absence of group differences in intrinsic hippocampal connectivity in our study. The evidence for increased intrinsic hippocampal connectivity in schizophrenia is mixed. A small number of studies have shown increased (12, 21) or decreased (22, 23) connectivity, but the majority have observed normal hippocampal connectivity (19, 24, 25, 27–29). ALFF is correlated with baseline CBF (48) and measures the amplitude of BOLD signal fluctuations in a single voxel, rather than the association between activation of voxels in different brain regions. It is therefore possible that connectivity between the hippocampus and other brain regions remains intact despite elevated hippocampal activity in patients. Both previous positive findings identified greater intrinsic hippocampal functional connectivity with the default mode network (12, 21). The hippocampus, however, is not always strongly connected with all regions in this network (18). The dissociation between ALFF and connectivity may result from weak overall hippocampal connectivity with the default mode network. Indeed, one recent study found that hippocampal-default mode network connectivity in schizophrenia patients was altered only with the precuneus (49). Our finding does not preclude hippocampal involvement in schizophrenia pathology. For example, hippocampal dysfunction has been linked to memory deficits in the disease (5). Alternatively, the hyperconnectivity observed in previous studies may have been driven by increased activity in other areas of the default mode network, not the hippocampus. Methodological differences may also have contributed to the failure to replicate increased hippocampal connectivity in schizophrenia. We instructed subjects to keep their eyes closed during the resting state scan, while the studies that found increased connectivity scanned subjects with eyes opened. Because connectivity is stronger when subjects have their eyes open (50), it is possible that this contributed to the different finding. However, it is unclear why this would differentially affect the patient and control groups.

Our findings support the hypothesis of increased intrinsic hippocampal activity in schizophrenia and are consistent with a translational study showing increased hippocampal CBV in patients and a mouse model of psychosis (11). Increased hippocampal ALFF is associated with a greater number of interictal epileptiform discharges in medial temporal lobe epilepsy (51), suggesting that ALFF is sensitive to the changes in glutamatergic and GABAergic signaling thought to drive hippocampal hyperactivity in schizophrenia (2–5). Although we did not find evidence that increased hippocampal ALFF impacts default mode network function, an intriguing alternative possibility is that increased hippocampal ALFF may lead to functional changes within the mesolimbic dopamine system. It has been proposed that elevated hippocampal activity drives positive symptoms of psychosis by increasing midbrain dopamine release through a loop involving the nucleus accumbens and ventral pallidum (52). One recent study found that altered connectivity between these regions in unmedicated schizophrenia patients was restored following anti-psychotic treatment (53). Future work should examine whether increased hippocampal ALFF is associated with dysconnectivity of this system and the corresponding link to psychotic symptoms.

We did not find evidence of increased hippocampal connectivity by varying the number of independent components identified in an analysis. Indeed, there was a trend for greater connectivity in controls compared to patients when using 60 components. One possible reason for the difference at a higher number of components could be greater resolution of data. The DMN component at 60 ICs may represent a subnetwork with a more specific hippocampal region than lower IC analyses (54). Although ICA and seed-based connectivity produce similar maps of the default mode network (55), the hippocampus may have the greatest connectivity with subregions of the default mode network, including the ventromedial prefrontal, parahippocampal, and posterior inferior parietal cortices rather than the core midline areas (18, 56, 57). It is possible that our 60-component analysis more correctly identified the medial temporal lobe subnetwork of the default mode network.

We conclude that inferences drawn from group ICA studies need to be viewed with caution. In group ICA, the entire group contributes to the estimated individual subject component maps, causing a violation of the assumption of independence and possibly a resulting error in statistical tests. Bootstrap or permutation methods applied before the ICA (i.e., re-computing the ICA for each bootstrap or permutation sample) may be required for accurate statistics. Different choices of which and how many independent components to use in the analysis resulted in different findings, raising doubts about the validity of this method to test the hypothesis of hippocampal hyperconnectivity in schizophrenia (58, 59). For component selection, automated methods that rely on template matching may need manual validation.

Given the limitations of *post hoc*, data-driven approaches, we also used a hypothesis-driven, seed-based approach that examined functional connectivity between the hippocampus and *a priori* defined DMN regions. This analysis yielded similar results; hippocampal connectivity with the DMN in schizophrenia patients was virtually identical to healthy control subjects. To our knowledge, this is the first time hippocampal functional connectivity in schizophrenia has been examined using both ICA (including group and individual ICA) and seed-based approaches. The convergence of findings across methods strongly argues against hippocampal hyperconnectivity, measured with RS-fMRI, as a biomarker for schizophrenia, and raises concerns about the usefulness of this method as an endpoint in clinical trials.

Although the primary aim of our study was the replication of previous findings (12, 33), assessment of chronic schizophrenia patients was a limitation of the present study. Hippocampal hyperactivity normalizes with treatment (17) and may have limited our ability to observe a difference in connectivity between patients and controls. However, it is unclear why treatment effects would influence connectivity and not ALFF. Future work should focus on examining hippocampal connectivity in the early stages of psychosis to minimize the influence of medication on activity and connectivity. While outside the scope of the current study, examination of hippocampal connectivity with individual brain regions rather than a broad system such as the default mode network may be a fruitful direction for future research. Hyperactivity of specific hippocampal subfields such as CA1 (16) or CA3 (3) may explain cognitive deficits in schizophrenia (1, 12). The resolution of our data was lower than that used in contrast-enhanced imaging studies and is not suitable to test subfield-specific hypotheses. Future studies should examine hippocampal activity using higher resolution imaging methods to identify the source of specific cognitive deficits in schizophrenia.

In summary, we found greater hippocampal ALFF but normal intrinsic hippocampal connectivity in chronic schizophrenia patients. Additionally, our data show that results of ICA studies can depend critically on algorithmic parameters. We conclude that ALFF may be a better marker than functional connectivity to identify individual and group differences in hippocampal function in schizophrenia.

## Conflict of Interest Statement

The authors declare that the research was conducted in the absence of any commercial or financial relationships that could be construed as a potential conflict of interest.

## Footnotes

^1^http://www.fil.ion.ucl.ac.uk/spm

^2^http://dbm.neuro.uni-jena.de/vbm

^3^http://mialab.mrn.org/software/gift/index.html

^4^http://www.fmrib.ox.ac.uk/fsl
